# Patient Outcomes With Use of Computed Tomography Angiography in Acute Ischemic Stroke and Transient Ischemic Attack: A Systematic Review and Meta-Analysis

**DOI:** 10.7759/cureus.8187

**Published:** 2020-05-18

**Authors:** Siying (Shari) Li, Aleksandar Trajkovski, Michael Siarkowski, Calvin Santiago, Kevin A Eng, Teruko Kishibe, Athithyan Umakanthan, Eddy Lang

**Affiliations:** 1 Emergency Medicine, University of British Columbia, Vancouver, CAN; 2 Family Medicine, Dalhousie University, Halifax, CAN; 3 Biomedical Technology, University of Calgary, Calgary, CAN; 4 Dentistry, University of British Columbia, Vancouver, CAN; 5 Neurology, University of Toronto, Toronto, CAN; 6 Radiology, Western University, London, CAN; 7 Scotiabank Health Sciences Library, St Michael's Hospital, Toronto, CAN; 8 Epidemiology and Public Health, Schulich School of Medicine & Dentistry, Western University, London, CAN; 9 Emergency Medicine, University of Calgary, Calgary, CAN

**Keywords:** stroke, ischemic stroke, transient ischemic attack, tia, computed tomography angiography, ct angiography, cta

## Abstract

Objectives

It remains uncertain whether computed tomography angiography (CTA) in ischemic strokes and transient ischemic attacks (TIAs) benefits patient outcomes beyond those eligible for endovascular therapy. We conducted a systematic review and meta-analysis of observational studies and randomized controlled trials (RCTs) investigating the use of CTA against other imaging modalities for recurrent stroke, mortality, disability, emergency department (ED) revisits, or changes in management in ischemic stroke and TIA. (PROSPERO: 349590)

Methods

MEDLINE, Embase, and CENTRAL were searched. We included studies evaluating CTA against non-CTA imaging modalities for outcomes of interest in ischemic stroke or TIA. Two reviewers extracted data and assessed study quality. Data were pooled by the generic inverse variance method. Heterogeneity was assessed using Cochran’s Q statistic and quantified by I^2^. Quality of the evidence was assessed by GRADE.

Results

We found 12 eligible cohort studies involving 17,481 patients, and no eligible RCTs. No changes were detected in recurrent stroke, mortality, or disability when CTA was compared against pooled imaging modalities, nor compared to non-contrast computed tomography (NCCT) alone. The evidence for each outcome was graded as low quality to very low quality.

Conclusions

CTA use was not associated with significant reductions in recurrent stroke, mortality, or disability in ischemic stroke and TIA patient compared with other imaging modalities. More high-quality studies are needed.

## Introduction

Stroke has a major global burden as a leading cause of mortality and morbidity worldwide. Increasingly, patients with stroke as well as transient ischemic attacks (TIAs) are receiving computed tomographic angiography (CTA) as part of the initial work up [1].

Recent guidelines reflect this trend: the 2018 Canadian Stroke Best Practice Guidelines recommend immediate CTA for acute stroke patients potentially eligible for endovascular therapy with A level evidence; and for very high risk patients presenting within 48 hours of non-disabling stroke or TIA, urgent CTA or magnetic resonance angiography (MRA) is recommended with B level evidence [2]. In the 2015 guidelines, for TIA patients not being considered for endovascular or thrombolytic therapy, CTA was recommended with C level evidence [3]. The American College of Emergency Physicians also recommend in their 2016 clinical policy for TIA that cervical vascular imaging (e.g., CTA) should be obtained when possible, with a grade C level recommendation [4].

There are several posited benefits to CTA use: CTA can be useful for secondary stroke prevention strategies such as carotid endarterectomy and stenting; it can help predict prognosis; and it can increase diagnostic yield for certain types of strokes [5]. Additionally, CTA has been used in recent endovascular trials to guide therapy, as reflected by the 2018 Canadian Stroke Best Practice Guidelines recommendation for patients presenting within acute stroke treatment windows [2].

However, use of CTA comes with costs to both the healthcare system and the patient. The radiation exposure of a head and neck CTA is around 5mSv, or two times the average annual background radiation dose worldwide [6]. Additionally, while the risk of contrast-induced nephropathy is low, this remains a necessary consideration in choosing CTA for patients presenting with stroke or TIA [6].

Given the potential risks, it is important to evaluate whether CTA use has an impact on patient-important outcomes, such as recurrent stroke and mortality. This is particularly pertinent where patients present outside of acute treatment time windows or criteria, and non-CTA imaging protocols are more commonly utilized. Therefore, we have conducted a systematic review and meta-analysis on the use of CTA compared with other imaging modalities in patients presenting with ischemic stroke or TIA for patient-important outcomes, with stratified analyses based on the type of stroke presentation. The primary outcome was recurrent stroke, and secondary outcomes included mortality, disability, emergency department (ED) revisits, and changes in medical or surgical management.

## Materials and methods

The study protocol was registered on PROSPERO (CRD42016039861). Study methods followed the Cochrane Handbook for Systematic Review of Interventions and data reporting conforms to Preferred Reporting Items for Systematic Reviews and Meta-Analyses (PRISMA) guidelines [7,8].

Literature search

TK searched MEDLINE, Embase, and the Cochrane Central Register of Controlled Trials through 7 March 2019 for eligible trials. Appendix Table 4 shows our detailed search strategy.

Study selection

We included observational cohort studies and randomized controlled trials (RCTs) evaluating use of CTA and recurrent stroke rate, mortality, disability, ED revisits, and changes in medical or surgical management in patients presenting acutely with ischemic strokes or TIAs. Only studies comparing head and neck CTA against a non-CTA control group were included. Studies using pediatric populations or confounding co-interventions with CTA exposure were also excluded. Studies were not limited based on language.

Data extraction

Results and study characteristics from eligible trials were double extracted by SSL and AT. Extracted characteristics include design, study setting, sample size, patient characteristics, duration of follow-up, control imaging modalities, and any criteria pertaining to use of CTA versus control imaging. Disability data was extracted and most commonly reported as risk ratios for favorable Modified Rankin Scale (mRS) scores of ≤ 2; where unavailable, risk ratios for mRS ≤ 1 or imputed risk ratios based on mean and standard deviation mRS scores were used instead [9, 10]. Methodological quality of eligible studies was also assessed by SSL and AT using the Newcastle-Ottawa Scale (NOS) for cohort studies, which awards up to nine points based on criteria pertaining to cohort selection, comparability of cohorts, and assessment of outcomes; a score of ≥ 6 was considered high quality [11]. RCTs were to be assessed by the Cochrane Risk of Bias tool [7]. Any disagreements between co-extractors in data extraction or quality assessment were reconciled by consensus.

Grading of the evidence

The Grading of Recommendations Assessment, Development, and Evaluation (GRADE) tool was used to assess the quality of evidence [12]. GRADE rates evidence as high, moderate, low, or very low quality. Observational cohort studies are graded as low-quality evidence by default, and can be further downgraded based on criteria pertaining to risk of bias (weight of studies showing risk of bias reflected by low NOS < 6), inconsistency (*I^2^* > 50% indicating substantial inter-study heterogeneity), indirectness (presence of factors limiting the generalizability of the results), imprecision (sample size less than the optimal information size and/or confidence interval including both appreciable benefit and appreciable harm), and publication bias (evidence of small-study effects).

Statistical methods

We used Review Manager version 5.3 (The Nordic Cochrane Centre, The Cochrane Collaboration, Copenhagen) for primary and sensitivity analyses.

Data were pooled using the generic inverse variance method with random-effects models and expressed as risk ratios (RR) with 95% CIs [7]. Where RR were unavailable, adjusted odds ratios (OR) were converted into RR using assumed control risks calculated by standard formulae, based on mean control group risks [7]. Where there were zero events in either group, we used the treatment arm continuity correction, which adds a factor of the reciprocal of the opposite cohort to each cell of the 2 x 2 table. This method has been found to be superior to the 0.5 continuity correction method, particularly in the case of unbalanced group sizes [13]. Furthermore, this method allows inclusion of studies with zero events in both cohorts, which is recommended to provide a conservative estimate of effect size. Where multiple comparisons were made within a single study, the comparator arm sample size was split between comparisons to mitigate unit-of-analysis error [7].

Inter-study heterogeneity was assessed using the Cochran’s Q statistic and quantified by the *I^2^* statistic. P-values < 0.10 were considered significant for heterogeneity, and an *I^2^* ≥ 50% was considered substantial [7].

To minimize heterogeneity in our analyses, studies comparing CTA with non-contrast computed tomography (NCCT) alone were meta-analysed together, and additional analyses were conducted comparing CTA against all pooled imaging comparators. Analyses for each outcome were stratified by stroke severity (either major stroke or minor stroke/TIA, defined as NIHSS ≥ 6 or < 6 respectively) [14]. Where NIHSS scores were not available, studies were stratified based on the authors’ classifications of major or minor stroke. For the one study where neither option was available, it was assumed for purposes of inclusion in stratification that the patient population had a median NIHSS ≥ 6 as the study evaluated only thrombolysis patients; this study was also removed in sensitivity analyses. Where possible, study data for major strokes, minor strokes, and TIAs were extracted separately to allow for direct comparisons. Supplementary analyses were conducted to segregate in-hospital versus long-term endpoints. Sensitivity analyses were additionally conducted, wherein all imputed data were removed, and wherein each study was systematically removed to assess the robustness of our pooled effect estimates. Due to the small number of studies in each analysis and the risk for erroneous results, publication bias analyses and subgroup analyses were not conducted [7, 15].

## Results

Study selection

**Figure 1:**
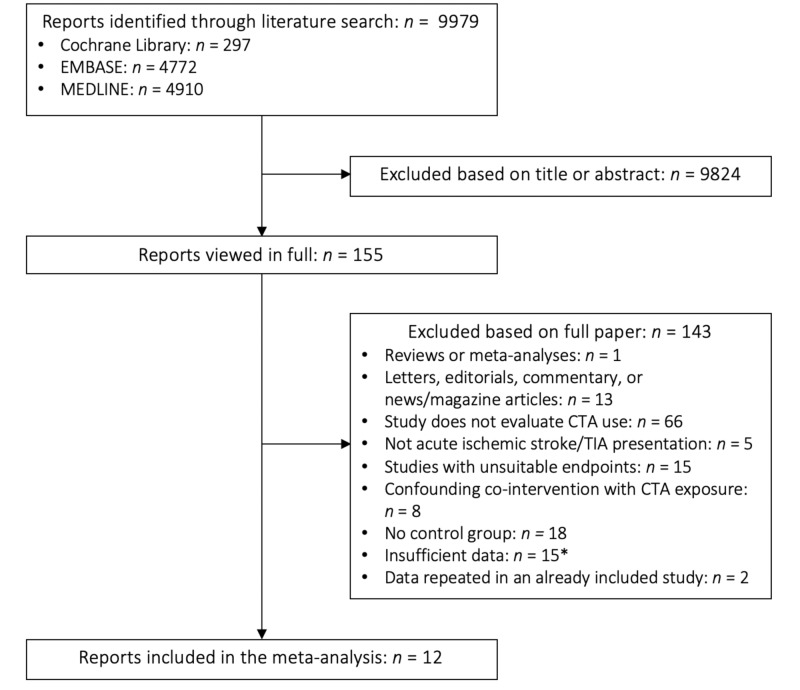
PRISMA Search Summary *We attempted to contact authors for all studies with insufficient data (e.g., conference abstracts). The ones for which we were unable to retrieve further data were ultimately excluded.

Figure 1 shows the study selection process. Our search identified 9979 reports, of which 9824 were excluded based on review of titles and abstracts. The remaining 155 papers were reviewed in full, of which 12 observational studies were included in our analyses [16-27].

Study characteristics

Table 1 summarizes the characteristics of included studies. In total, 17481 patients with a median age of 69 years were included in this analysis. All studies used NCCT or magnetic resonance imaging (MRI) as part of initial workup. Eleven studies compared CTA against NCCT alone, and three studies performed comparisons of CTA against MRI, MRA, Doppler’s ultrasound (DUS), and/or computed tomography perfusion (CTP). We found no studies evaluating ED revisits or changes in management as outcomes. All studies except one were assessed as high quality by the Newcastle Ottawa Scale (NOS ≥ 6).

**Table 1:** Study Characteristics NIHSS = NIH Stroke Scale; MCA = Middle cerebral artery; NOS = Newcastle Ottawa Scale; CTA = Computed tomography angiography; NCCT = Non-contrast computed tomography; DUS = Doppler's ultrasound; MRI = Magnetic resonance imaging; CTP = Computed tomography perfusion; MRA = Magnetic resonance angiography. All studies were cohort design. * Bill et al. (2017) had declared conflicts of interest; no other studies declared conflicts of interest. † Mean ± SD data were used as available; where unavailable, mean, median (IQR), or median (range) was used. For Hefzy et al. (2013), only median data was available for presenting NIHSS. Studies where the data was not available are demarcated by N/A. ‡ All patients across cohorts received NCCT head. § All patients across cohorts received NCCT and/or MRI. || Reflects symptomatic intracerebral hemorrhage, only included in sensitivity analyses for recurrent stroke outcome. ¶ Multivariate logistic regression adjustments. # Only adjusted for disability outcome. ** Only adjusted for mortality outcome. †† Matched design. ‡‡ Disability outcome lower by 1 point due to failure to demonstrate that outcome was not present at start of study. §§ Disability outcome higher by 1 point (+2 points due to comparability of cohorts on the basis of design or analysis and -1 point due to failure to demonstrate that outcome was not present at start of study). |||| Disability outcome lower by 2 points due to lack of comparability of cohorts on the basis of design or analysis. ¶¶ Only adjusted in separate comparator analyses (not as pooled analysis).

Study, year	N	Age †	Presenting NIHSS †	Population	Setting	Intervention	Comparator	Outcomes	Design	Follow-up	Controlled factors	NOS	Country
Atchaneeyasakul et al., 2017 [16]	34	68	16	Anterior circulation occlusion stroke receiving endovascular therapy	Stroke unit	CTA	NCCT ^‡^	Disability	Prospective cohort	In-hospital	-	6	USA
Aulicky et al., 2009 [17]	241	69	14 (4)	Ischemic stroke receiving thrombolytics	Stroke unit	CTA	NCCT ^‡^	Mortality, disability	Retrospective cohort	3 mo	-	6	Czech Republic
Bill et al., 2017* [18]	684	73 (21)	4 (10)	Ischemic stroke	Stroke unit/ICU	CTA	NCCT ^‡^	Mortality, recurrent stroke, disability	Retrospective cohort	12 mo (disability 3 mo)	Age, stroke onset, Cr, pre-hospital mRS, admission NIHSS, glucose, temperature, early ischemic/chronic CT changes^¶^	8	Switzerland
Dzialowski et al., 2012 [19]	1205	70	13 (5)	Ischemic stroke	Stroke unit	CTA	NCCT ^‡^	Disability	Retrospective cohort	3 mo	Age, baseline NIHSS, diabetes mellitus, onset-to-treatment time, pre-tPA antiplatelet therapy ^¶^	6 ^‡‡^	Canada
Eichel et al., 2014 [20]	73	72	15	Proximal MCA occlusion stroke receiving thrombolytics	ED followed by stroke unit	CTA	NCCT ^‡^	Mortality, recurrent stroke ^||^, disability	Retrospective cohort	3 mo	Age, admission NIHSS, symptomatic intracerebral hemorrhage ^¶ #^	4 ^§§^	Israel
Garcia Pastor et al., 2014 [21]	244	70 ± 13	14 (5)	Ischemic stroke receiving thrombolytics	Stroke unit	CTA	NCCT, DUS ^‡^	Mortality, recurrent stroke ^||^, disability	Prospective cohort	In-hospital (disability 3 mo)	-	6 ^‡‡^	Spain
Hefzy et al., 2013 [22]	727	67 ± 14	4	Ischemic stroke/TIA	Stroke unit	CTA	NCCT and/or MRI ^§^	Mortality, recurrent stroke	Prospective cohort	12 mo	-	7	USA
McDonald et al., 2014 [23]	12429	72 (12)	N/A	Ischemic stroke receiving thrombolytics	Hospital admission for stroke	CTA	NCCT +/- CTP or MRI ^‡^	Mortality	Prospective cohort	In-hospital (median 5 d)	Age, sex, race, admission status, admission source, payer, Charlson score, thrombolysis timing, provider specialty, hospital characteristics ^¶ ¶¶^	8	USA
Radecki et al., 2015 [24]	1014	68 (12)	11 (7)	Ischemic stroke receiving thrombolytics	Stroke unit	CTA	NCCT ^‡^	Mortality, recurrent stroke ^||^	Retrospective cohort	In-hospital	Age, CAD/MI, infarct ^¶ **^	8 ^||||^	USA
Torres-Mozqueda et al., 2008 - Major Stroke [25]	58	67 ± 15	N/A (major stroke)	Ischemic stroke (major)	ED	CTA + NCCT	MRI + MRA	Mortality	Prospective cohort	In-hospital (mean 12 d)	-	7	USA
Torres-Mozqueda et al., 2008 - Minor Stroke [25]	172	69 ± 8	N/A (minor stroke)	Ischemic stroke (minor)	ED	CTA + NCCT	MRI + MRA	Mortality	Prospective cohort	In-hospital (mean 3 d)	-	7	USA
Vagal et al., 2016 - Endovascular therapy [26]	369	69 (10)	17 (4)	Ischemic stroke NIHSS ≥ 10 receiving endovascular therapy following IV tPA	Multi-centre	CTA	NCCT ^‡^	Disability	Prospective cohort	3 mo	Age, baseline NIHSS, onset-to-treatment time ^¶^	8	USA and Canada
Vagal et al. 2016 - IV thrombolysis only [26]	189	69 (10)	17 (4)	Ischemic stroke NIHSS ≥ 10 receiving IV thrombolytics	Multi-centre	CTA	NCCT ^‡^	Disability	Prospective cohort	3 mo	Age, baseline NIHSS, onset-to-treatment time ^¶^	8	USA and Canada
Veronel et al., 2008 [27]	42	62	12 (2-12)	Ischemic stroke receiving thrombolytics	Stroke unit	CTA	NCCT ^‡^	Disability	Prospective cohort	In-hospital (mean 5 d)	Localization of infarct, dense artery sign in NCCT, admission NIHSS and mRS, onset-to-treatment time ^††^	7	Germany

Recurrent stroke

**Figure 2:**
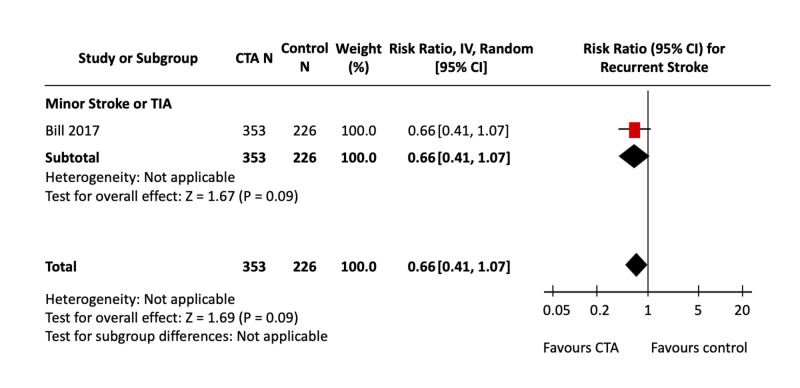
CTA vs NCCT for Recurrent Stroke Forest Plot The pooled effect estimates (diamonds) are shown for studies in minor stroke/TIA patients and the total. There were no studies in major stroke. Data are expressed as risk ratios with 95% confidence intervals, using generic inverse-variance random-effects models. Interstudy heterogeneity was tested using the Cochran Q statistic (chi-square) at a significance level of P < 0.10 and quantified using the *I^2^* statistic. CTA: Computed tomography angiography; NCCT: Non-contrast computed tomography; TIA: Transient ischemic attack.

**Figure 3:**
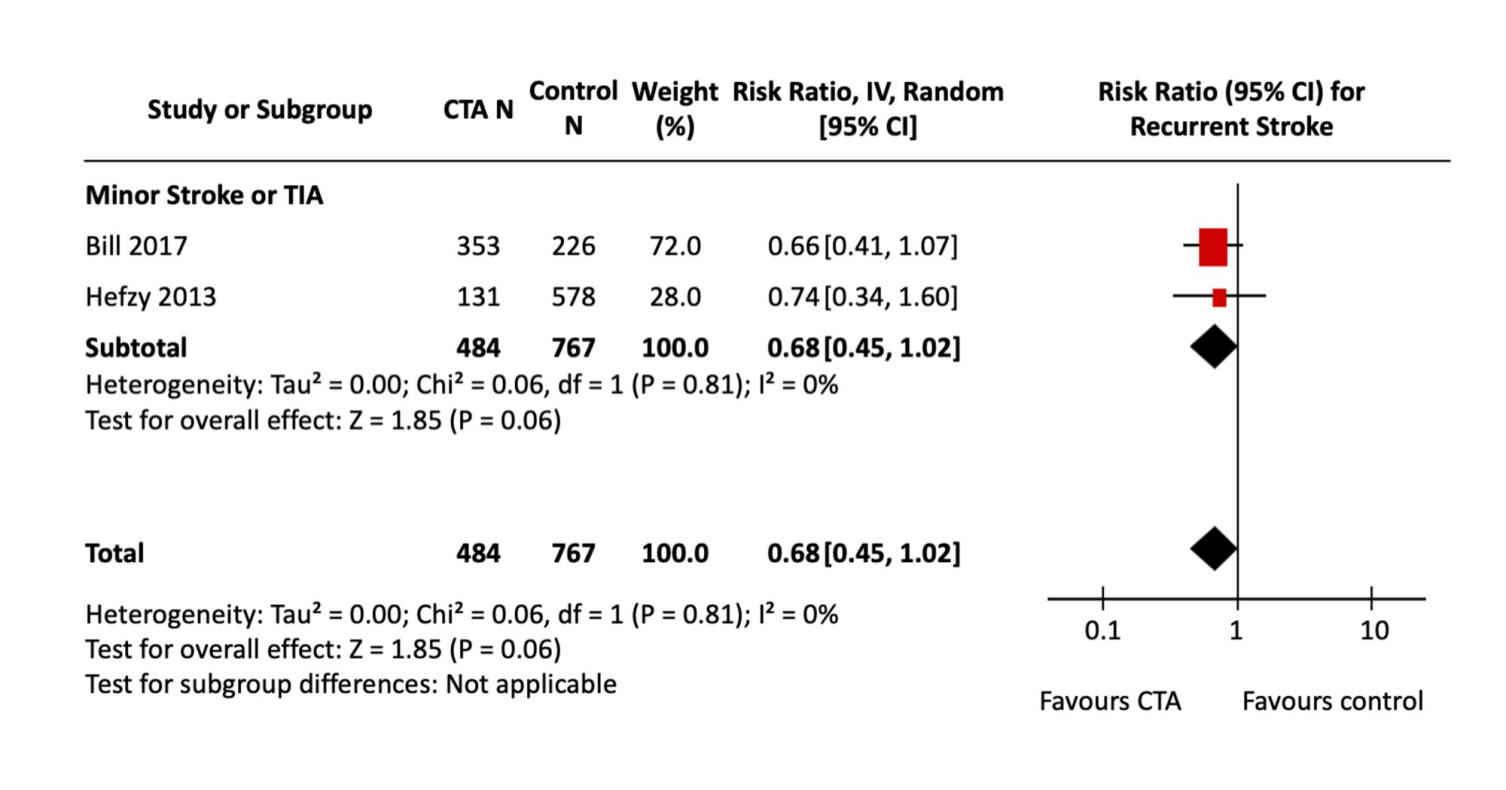
CTA vs non-CTA for Recurrent Stroke Forest Plot The pooled effect estimates (diamonds) are shown for studies in minor stroke/TIA patients and the total. There were no studies in major stroke. Data are expressed as risk ratios with 95% confidence intervals, using generic inverse-variance random-effects models. Interstudy heterogeneity was tested using the Cochran Q statistic (chi-square) at a significance level of P < 0.10 and quantified using the *I*^2^ statistic. CTA: Computed tomography angiography; TIA: Transient ischemic attacks.

Figure 2 shows the pooled effect estimate of CTA compared with NCCT alone for recurrent stroke in patients presenting with ischemic stroke and TIA. Figure 3 shows the effect of CTA against all non-CTA comparators. Only two studies, both in patients presenting with minor stroke or TIA, evaluated recurrent stroke as an outcome; both studies looked at only long-term recurrent stroke risks. Bill et al. used NCCT as the comparator imaging modality, and Hefzy et al. used either NCCT or MRI in all patients. The calculated assumed control risk was 8%, which falls within the range of 4-14% seen in the literature for recurrent stroke at up to one-year follow-up [28, 29].

The comparison of CTA against NCCT alone, which included the one study by Bill et al., was not significantly associated with decreased recurrent stroke [RR, 0.66 (95% CI, 0.41 to 1.07), P = 0.09]; nor was the comparison of CTA against all non-CTA imaging modalities [RR, 0.68 (95% CI, 0.45 to 1.02), P = 0.06; *I^2^* = 0%]. Heterogeneity for the CTA vs non-CTA analysis was non-significant; for the single NCCT comparison, heterogeneity was not applicable.

Additional sensitivity analyses were conducted to include major stroke studies which evaluated symptomatic intracranial hemorrhage (sICH) following thrombolytics as a secondary stroke outcome. Data for combined and in-hospital only outcomes are presented separately in Appendix Figures 8-11.

Mortality

**Figure 4:**
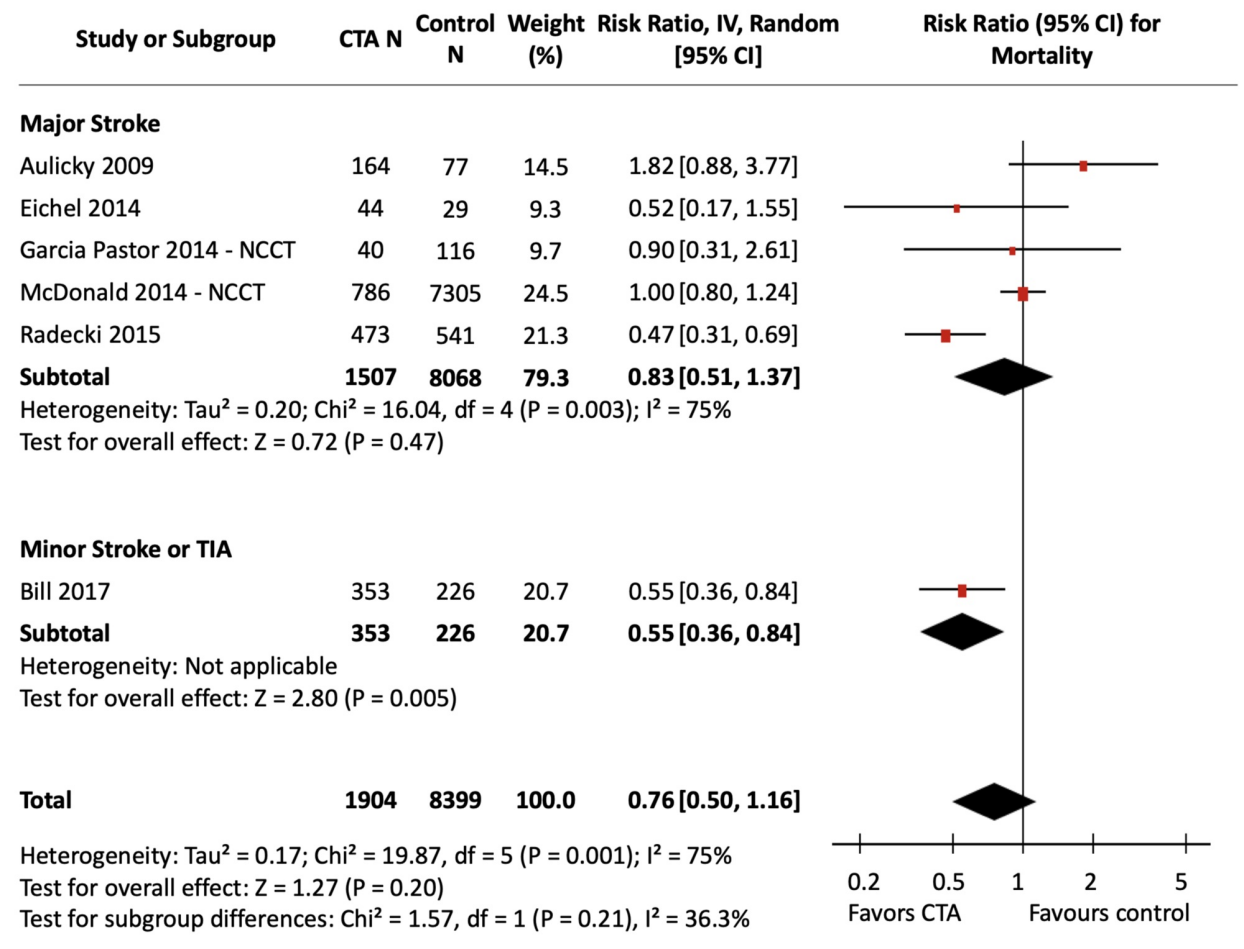
CTA vs NCCT for Mortality Forest Plot Three pooled effect estimates (diamonds) are shown: one each for studies in major stroke patients, minor stroke/TIA patients, and their combination (total). Data are expressed as risk ratios with 95% confidence intervals, using generic inverse-variance random-effects models. Interstudy heterogeneity was tested using the Cochran Q statistic (chi-square) at a significance level of P < 0.10 and quantified using the *I^2^* statistic. CTA: Computed tomography angiography; NCCT: Non-contrast computed tomography.

**Figure 5:**
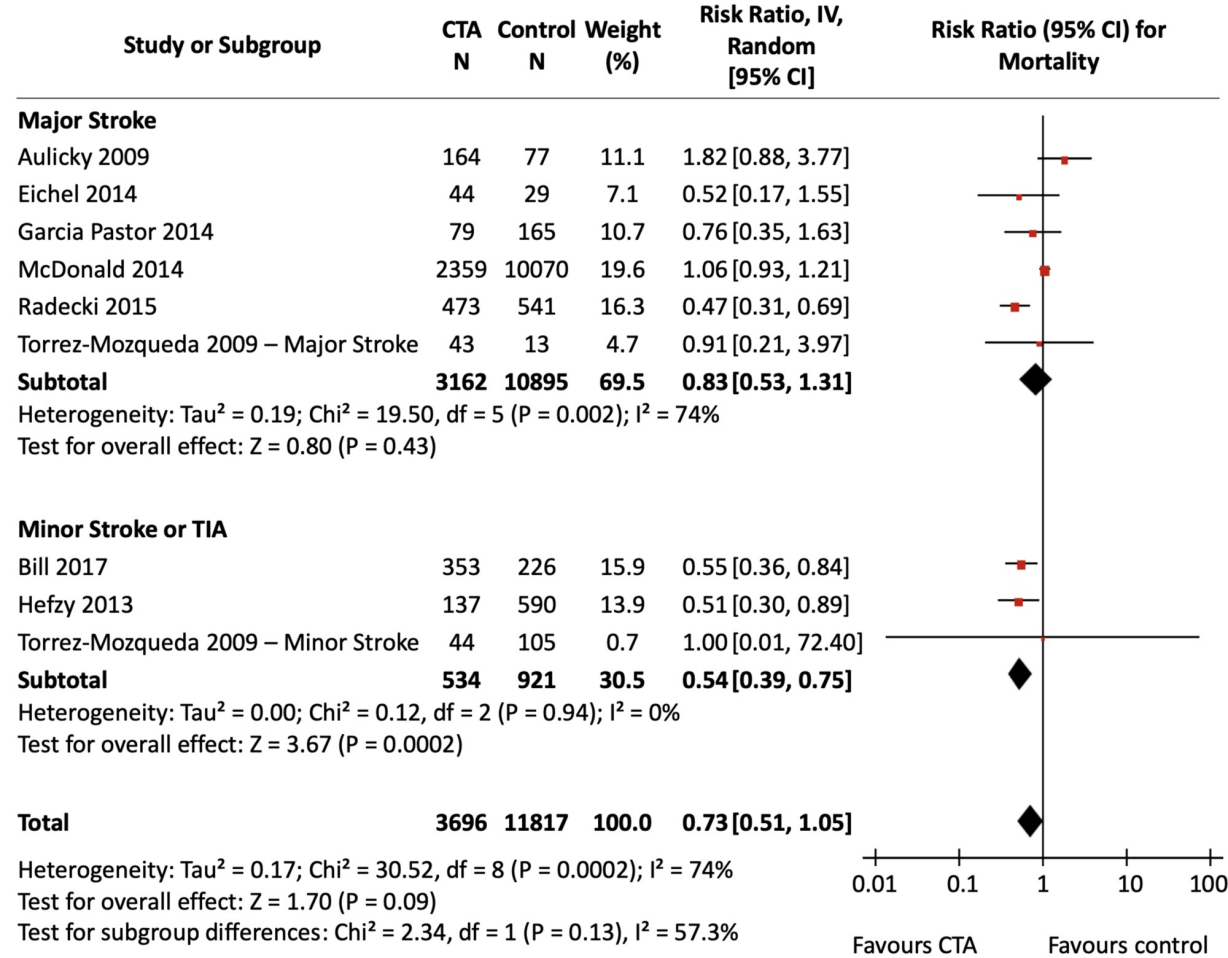
CTA vs non-CTA for Mortality Forest Plot Three pooled effect estimates (diamonds) are shown: one each for studies in major stroke patients, minor stroke/TIA patients, and their combination (total). Data are expressed as risk ratios with 95% confidence intervals, using generic inverse-variance random-effects models. Interstudy heterogeneity was tested using the Cochran Q statistic (chi-square) at a significance level of P < 0.10 and quantified using the *I^2^* statistic. CTA: Computed tomography angiography; TIA: Transient ischemic attack.

Figure 4 presents the meta-analysed effect estimate of CTA use compared with NCCT alone for mortality in ischemic stroke and TIA, stratified by stroke severity. Figure 5 shows the stratified effect of CTA against all non-CTA comparators. Comparator imaging modalities were limited to NCCT for six studies; other comparator imaging modalities included DUS, CTP, MRI, and MRA. The calculated assumed control risk used for in-hospital mortality was 10%, which correlates with previously published data [30]; the calculated assumed control risk for long-term mortality was 18%, which is comparable to previously observed rates of mortality up to one year following ischemic stroke [30]. Data for in-hospital and long-term outcomes are presented separately in Appendix Figures 12-15.

The pooled analysis did not detect a significant difference in mortality with CTA compared to NCCT alone [RR, 0.76 (95% CI, 0.50 to 1.16), P = 0.20]. There was significant and substantial interstudy heterogeneity (*I^2^* = 75%, P_het_ = 0.001). Stratified analyses did not show a significant association of CTA with reduced mortality in the major stroke subgroup (P = 0.47) but did show a significant association in the minor stroke/TIA subgroup (P = 0.005) which included only the study by Bill et al.. The test for differences between subgroups was non-significant (P = 0.21).

The meta-analyzed comparison of CTA against all non-CTA imaging modalities also was not associated with a significant difference in mortality, and again heterogeneity was both significant and substantial [RR, 0.73 (95% CI, 0.51 to 1.05), P = 0.09; *I^2^* = 74%, P_het_ = 0.0002]. Stratified analyses showed a significant association of CTA with reduced mortality in the minor stroke/TIA group without heterogeneity [RR, 0.54 (95% CI, 0.39 to 0.75), P = 0.0002; *I^2^* = 0%, P_het_ = 0.94]. There was no association of CTA use with changes in mortality in the major stroke group (P = 0.43). The test for subgroup differences was non-significant (P = 0.13).

In sensitivity analyses, removal of the study by McDonald et al. resulted in an overall significant decrease in mortality with CTA use compared to non-CTA imaging modalities (P = 0.008) and resolved heterogeneity (*I^2^* = 40%, P_het_ = 0.11). Removal of the study by Aulicky et al. also resulted in a significant decrease in mortality with CTA use compared to non-CTA imaging modalities (P = 0.03) without impacting heterogeneity. Sensitivity analyses did not alter comparisons between CTA and NCCT alone.

Disability

**Figure 6:**
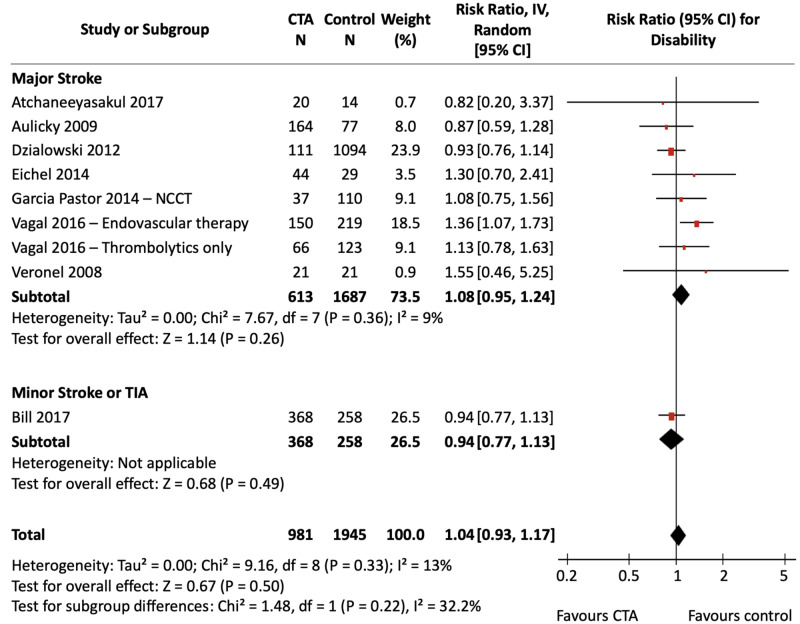
CTA vs NCCT for Disability Forest Plot Three pooled effect estimates (diamonds) are shown: one each for studies in major stroke patients, minor stroke/TIA patients, and their combination (total). Data are expressed as risk ratios with 95% confidence intervals, using generic inverse-variance random-effects models. Interstudy heterogeneity was tested using the Cochran Q statistic (chi-square) at a significance level of P < 0.10 and quantified using the *I^2^* statistic. CTA: Computed tomography angiography; NCCT: Non-contrast computed tomography; TIA: Transient ischemic attack.

**Figure 7:**
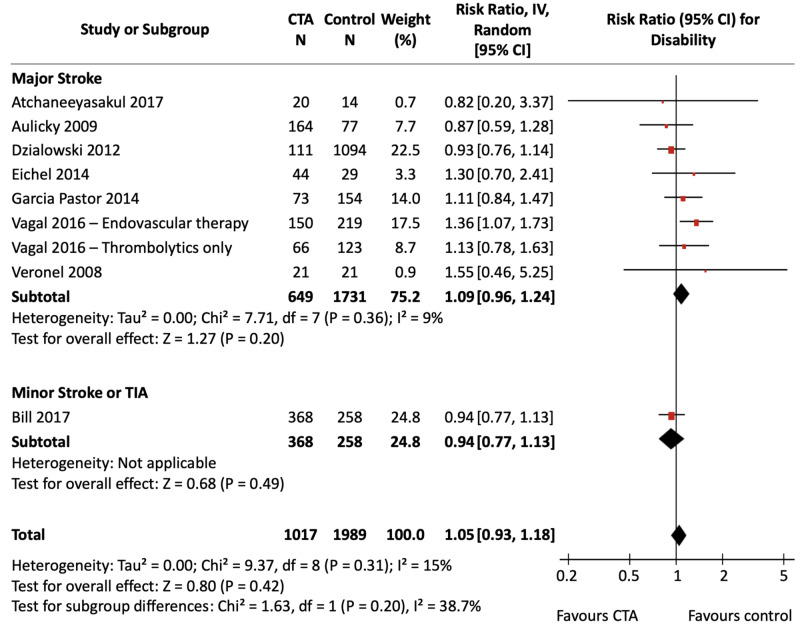
CTA vs non-CTA for Disability Forest Plot Three pooled effect estimates (diamonds) are shown: one each for studies in major stroke patients, minor stroke/TIA patients, and their combination (total). Data are expressed as risk ratios with 95% confidence intervals, using generic inverse-variance random-effects models. Interstudy heterogeneity was tested using the Cochran Q statistic (chi-square) at a significance level of P < 0.10 and quantified using the *I^2^* statistic. CTA: Computed tomography angiography; TIA: Transient ischemic attack.

Figure 6 shows the pooled effect estimate of CTA compared with NCCT alone for disability in patients presenting with ischemic stroke and TIA, stratified by stroke severity. Figure 7 shows the effect of CTA against all non-CTA comparators. All studies used NCCT alone as the comparator imaging modality except for Garcia Pastor et al., which also used DUS. The calculated assumed control risk for a mRS ≤ 2 was 46%, which correlates with previously published rates of favourable mRS three months post-stroke [30]. Data for in-hospital and long-term outcomes are presented separately in Appendix Figures 16-19.

The pooled analyses did not detect a significant difference in disability with CTA use compared to NCCT alone [RR, 1.04 (95% CI, 0.93 to 1.17), P = 0.50; *I^2^* = 13%], nor compared to pooled non-CTA imaging modalities [RR, 1.05 (95% CI, 0.93 to 1.18), P = 0.42; *I^2^* = 15%]. Stratified analyses did not show a significant association of CTA with reduced disability in either the major stroke groups or the minor stroke/TIA groups. Heterogeneity was non-significant for both analyses. Sensitivity analyses did not change results.

GRADE assessment

GRADE was used to assess the overall quality of evidence for CTA use with each of our outcomes.

Table 2 shows a summary of the GRADE assessments for the comparison of CTA versus NCCT for each recurrent stroke, mortality, and disability. The evidence for disability was rated low quality, the default level for observational studies. The evidence for recurrent stroke was downgraded to very low quality due to evidence of serious imprecision. The evidence for mortality was also downgraded to a grade of very low quality, due to evidence of serious inconsistency.

**Table 2:** GRADE Table for CTA vs NCCT CI: Confidence interval; RR: Risk ratio; CTA: Computed tomography angiography; NCCT: Non-contrast computed tomography. ^a^ Our sample size of 579 did not meet the optimal information size of 2699 required to detect a 25% change in recurrent stroke rate. ^b^ Individual study risk ratios were derived from adjusted odds ratios where given, resulting in pooled risk ratios that do not directly reflect pooled cohort proportions. ^c^ The I-squared value for the pooled analysis was 75%, with a P-value of 0.001. All individual study confidence intervals overlapped with the pooled effect estimate.

Quality assessment							No. of patients		Effect		Quality
No. of studies	Study design	Risk of bias	Inconsistency	Indirectness	Imprecision	Other considerations	CTA	Control	Relative (95% CI)	Absolute (95% CI)	
Recurrent Stroke Rate											
1	Observational studies	Not serious	Not serious	Not serious	Serious ^a^	None	48/353 (13.6%)	21/226 (9.3%)	RR 0.66 (0.41 to 1.07) ^b^	32 fewer per 1,000 (from 55 fewer to 7 more)	⨁◯◯◯ VERY LOW
Mortality											
6	Observational studies	Not serious	Serious ^c^	Not serious	Not serious	None	189/1904 (9.9%)	884/8399 (10.5%)	RR 0.76 (0.50 to 1.16) ^b^	25 fewer per 1,000 (from 53 fewer to 17 more)	⨁◯◯◯ VERY LOW
Disability											
9	Observational studies	Not serious	Not serious	Not serious	Not serious	None	378/981 (38.5%)	883/1945 (45.4%)	RR 1.04 (0.93 to 1.17) ^b^	18 more per 1,000 (from 32 fewer to 77 more)	⨁⨁◯◯ LOW

Table 3 shows a summary of the GRADE assessments for the comparison of CTA versus pooled non-CTA imaging modalities for each outcome. The evidence for disability was rated low quality by default. The evidence for recurrent stroke and mortality was again downgraded to very low quality due to evidence of serious imprecision and serious inconsistency, respectively.

**Table 3:** GRADE Table for CTA vs non-CTA CI: Confidence interval; RR: Risk ratio; CTA: Computed tomography angiography. ^a^ Our sample size of 1288 did not meet the optimal information size of 2699 required to detect a 25% change in recurrent stroke rate. ^b^ Individual study risk ratios were derived from adjusted odds ratios where given, resulting in pooled risk ratios that do not directly reflect pooled cohort proportions. ^c^ The *I^2^* value for the pooled analysis was 74%, with a P-value of 0.0002. All individual study confidence intervals overlapped with the pooled effect estimate.

Quality assessment							No. of patients		Effect		Quality
No. of studies	Study design	Risk of bias	Inconsistency	Indirectness	Imprecision	Other considerations	CTA	Control	Relative (95% CI)	Absolute (95% CI)	
Recurrent Stroke Rate											
2	Observational studies	Not serious	Not serious	Not serious	Serious ^a^	None	55/484 (11.4%)	63/804 (7.8%)	RR 0.68 (0.45 to 1.02) ^b^	25 fewer per 1,000 (from 2 more to 43 fewer)	⨁◯◯◯ VERY LOW
Mortality											
9	Observational studies	Not serious	Serious ^c^	Not serious	Not serious	None	373/3696 (10.1%)	1229/11817 (10.4%)	RR 0.73 (0.51 to 1.05) ^b^	28 fewer per 1,000 (from 5 more to 51 fewer)	⨁◯◯◯ VERY LOW
Disability											
9	Observational studies	Not serious	Not serious	Not serious	Not serious	None	397/1017 (39.0%)	902/1989 (45.3%)	RR 1.05 (0.93 to 1.18) ^b^	23 more per 1,000 (from 32 fewer to 82 more)	⨁⨁◯◯ LOW

## Discussion

To our knowledge, this is the first systematic review and meta-analysis evaluating CTA use against alternative imaging strategies for patient-important outcomes in stroke and TIA. Our analyses of 12 studies including 17481 subjects failed to detect significant changes in recurrent stroke, mortality, or disability with CTA use in acute ischemic stroke and TIA compared with pooled alternative imaging protocols and compared with NCCT alone. We found no studies assessing changes in management or ED revisits for ischemic stroke or TIA.

Recent studies and guidelines have recommended use of CTA for acute stroke patients; in particular, patients with major strokes who may be candidates for endovascular therapy can benefit from CTA as part of the initial work-up [2-4]. CTA is fast, accurate, and relatively accessible from the ED, making it ideal for selecting those patients who are eligible for endovascular therapy with minimal time delays. Additionally, CTA can be useful for secondary stroke prevention. Imaging of the carotids allows for referral of patients with high grade stenosis for carotid endarterectomy, which in turn provides significant risk reduction for recurrent stroke [2].

While we did not find a significant overall reduction in recurrent stroke, mortality, or disability with CTA use, our results were limited by the small number of studies in our analyses. We did however find a non-significant trend towards reduced recurrent stroke with CTA use compared to each NCCT alone and pooled non-CTA imaging modalities in minor stroke/TIA studies (P = 0.09 and P = 0.06, respectively). Additionally, we found a significant reduction in mortality for minor stroke/TIA with CTA use compared to each NCCT alone and pooled non-CTA imaging modalities (P = 0.005 and P = 0.0002, respectively). Together, these results suggest that CTA may have a role in reduced mortality in minor stroke/TIA, plausibly through secondary prevention of recurrent stroke. It should however be noted that the comparison of CTA against NCCT in minor stroke/TIA included only one study for both recurrent stroke and mortality.

The decreased mortality found in minor stroke/TIA was not seen in the major stroke group, but the major stroke group also contained significant and substantial heterogeneity that persisted despite separating analyses by duration of outcomes (see Appendix Figures 12-15). Considering the evolving management and technology around major strokes over the past decade, it is perhaps unsurprising that there would be such heterogeneity in outcomes.

Limitations

Our systematic review and meta-analysis has important limitations. First, we were limited by the small number of studies, which restricts our ability to perform meta-regression subgroup analyses [7]. This prevented us from meaningfully comparing different imaging modalities as control groups, and in particular limits our ability to compare CTA with DUS in the context of secondary prevention. However, we were able to perform separate analyses for studies using NCCT alone as the comparator, and to stratify our analyses based on stroke severity, in order to provide a more detailed analysis of possible contributing factors. Unfortunately, no data was provided on high, moderate, or low risk TIAs to further characterize these groups. Second, the significance of our results varied through sensitivity analyses, and removal of certain individual studies resulted in a significant pooled effect estimate for the comparison of CTA to pooled non-CTA imaging modalities in mortality. This is not entirely surprising given the near-significance of this analysis (P = 0.09), and suggests that additional studies may provide a more robust pooled effect estimate. Third, our mortality analyses had significant and substantial heterogeneity, which was neither explained by stratified analyses nor by sensitivity analyses, resulting in a downgrade for inconsistency. Additionally, our recurrent stroke analyses did not meet the optimal information size, resulting in downgrades for imprecision. Finally, because we found only observational data for our outcomes, effects from confounding cannot be ruled out. Approximately half of our included studies adjusted for confounding factors including age, baseline NIHSS and other criteria. Nonetheless, due to the observational nature of the data, all our outcomes were graded as low-quality evidence by default, and further downgraded where indicated.

## Conclusions

Our analyses found no significant changes in recurrent stroke, mortality, or disability with CTA use for patients presenting with acute ischemic stroke and TIA, compared to NCCT alone as well as compared to pooled non-CTA imaging protocols. No data was found for changes in management or ED revisits. Our results were limited by the observational data and the small number of studies. The disability outcome was graded as low-quality evidence, while the mortality and recurrent stroke outcomes were downgraded to very low quality for inconsistency and imprecision, respectively. More high-quality studies are needed to elucidate the role of CTA use in patient-important outcomes, particularly for patients presenting with minor strokes and TIAs.

## Figures and Tables

**Figure 8:**
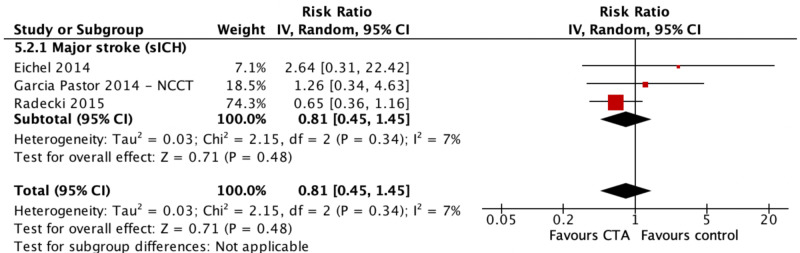
Recurrent Stroke for CTA vs NCCT, in-hospital outcomes sensitivity analysis The pooled effect estimates (diamonds) are shown for studies in major stroke patients and the total. There were no studies in minor stroke/TIA. Data are expressed as risk ratios with 95% confidence intervals, using generic inverse-variance random-effects models. Interstudy heterogeneity was tested using the Cochran Q statistic (chi-square) at a significance level of P < 0.10 and quantified using the *I^2^* statistic. CTA: Computed tomography angiography; NCCT: Non-contrast computed tomography; TIA: Transient ischemic attack.

**Figure 9:**
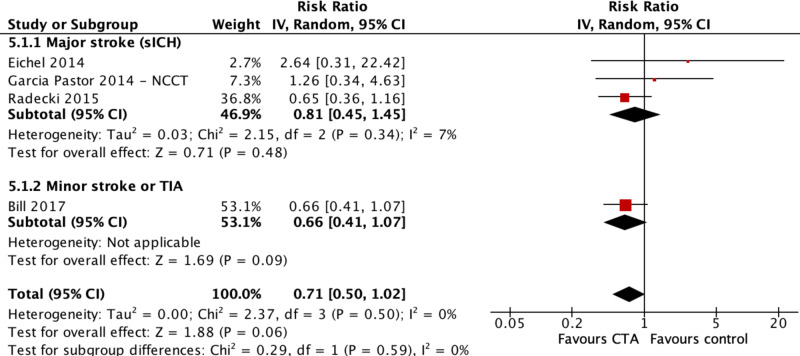
Recurrent Stroke for CTA vs NCCT, combined outcomes sensitivity analysis Three pooled effect estimates (diamonds) are shown: one each for studies in major stroke patients (sICH), minor stroke/TIA patients, and their combination (total). Data are expressed as risk ratios with 95% confidence intervals, using generic inverse-variance random-effects models. Interstudy heterogeneity was tested using the Cochran Q statistic (chi-square) at a significance level of P < 0.10 and quantified using the *I*^2^ statistic. CTA: Computed tomography angiography; NCCT: Non-contrast computed tomography; TIA: Transient ischemic attack.

**Figure 10:**
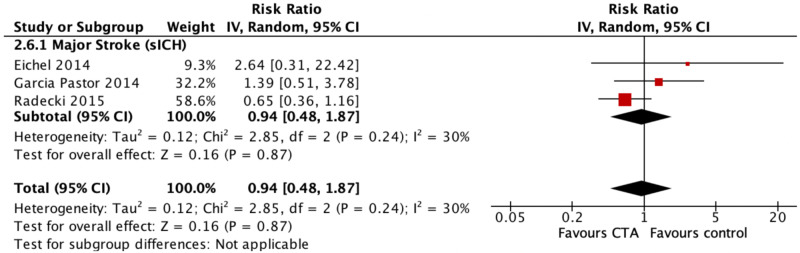
Recurrent Stroke for CTA vs non-CTA, in-hospital outcomes sensitivity analysis The pooled effect estimates (diamonds) are shown for studies in major stroke patients and the total. There were no studies in minor stroke/TIA. Data are expressed as risk ratios with 95% confidence intervals, using generic inverse-variance random-effects models. Interstudy heterogeneity was tested using the Cochran Q statistic (chi-square) at a significance level of P < 0.10 and quantified using the *I^2^* statistic. CTA: Computed tomography angiography; TIA: Transient ischemic attack.

**Figure 11:**
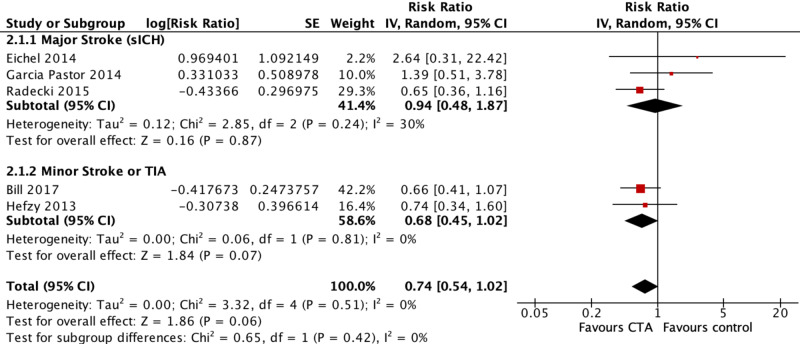
Recurrent Stroke for CTA vs non-CTA, combined outcomes sensitivity analysis Three pooled effect estimates (diamonds) are shown: one each for studies in major stroke patients (sICH), minor stroke/TIA patients, and their combination (total). Data are expressed as risk ratios with 95% confidence intervals, using generic inverse-variance random-effects models. Interstudy heterogeneity was tested using the Cochran Q statistic (chi-square) at a significance level of P < 0.10 and quantified using the *I*^2^ statistic. CTA: Computed tomography angiography; TIA: Transient ischemic attack.

**Figure 12:**
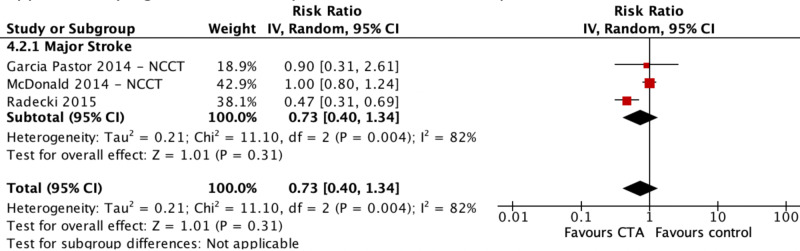
Mortality for CTA vs NCCT, in-hospital outcomes The pooled effect estimates (diamonds) are shown for studies in major stroke patients and the total. There were no studies in minor stroke/TIA. Data are expressed as risk ratios with 95% confidence intervals, using generic inverse-variance random-effects models. Interstudy heterogeneity was tested using the Cochran Q statistic (chi-square) at a significance level of P < 0.10 and quantified using the *I^2^* statistic. CTA: Computed tomography angiography; NCCT: Non-contrast computed tomography; TIA: Transient ischemic attack.

**Figure 13:**
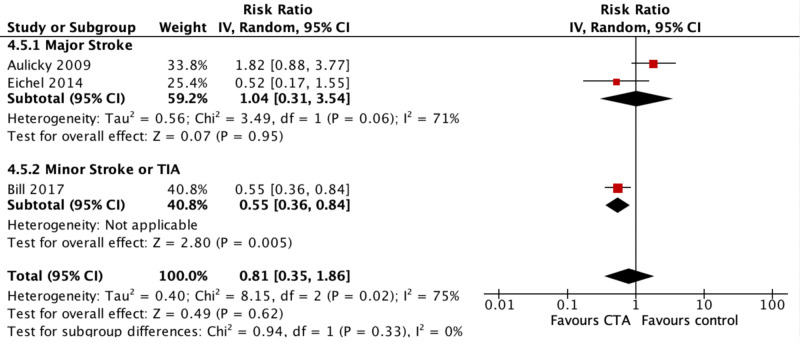
Mortality for CTA vs NCCT, long-term outcomes Three pooled effect estimates (diamonds) are shown: one each for studies in major stroke patients, minor stroke/TIA patients, and their combination (total). Data are expressed as risk ratios with 95% confidence intervals, using generic inverse-variance random-effects models. Interstudy heterogeneity was tested using the Cochran Q statistic (chi-square) at a significance level of P < 0.10 and quantified using the *I^2^* statistic. CTA: Computed tomography angiography; NCCT: Non-contrast computed tomography; TIA: Transient ischemic attack.

**Figure 14:**
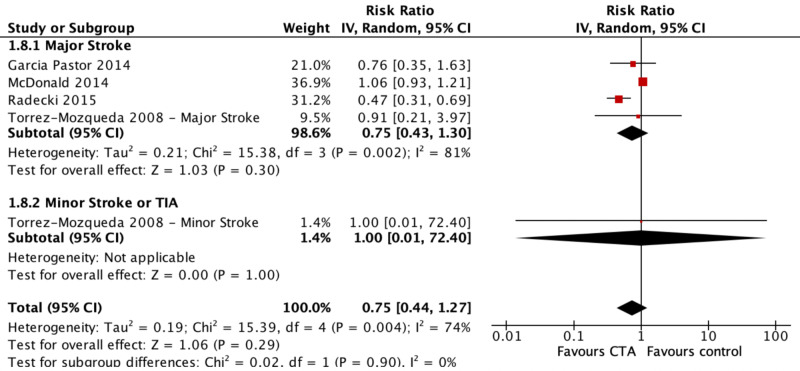
Mortality for CTA vs non-CTA, in-hospital outcomes Three pooled effect estimates (diamonds) are shown: one each for studies in major stroke patients, minor stroke/TIA patients, and their combination (total). Data are expressed as risk ratios with 95% confidence intervals, using generic inverse-variance random-effects models. Interstudy heterogeneity was tested using the Cochran Q statistic (chi-square) at a significance level of P < 0.10 and quantified using the *I^2^* statistic. CTA: Computed tomography angiography; TIA: Transient ischemic attack.

**Figure 15:**
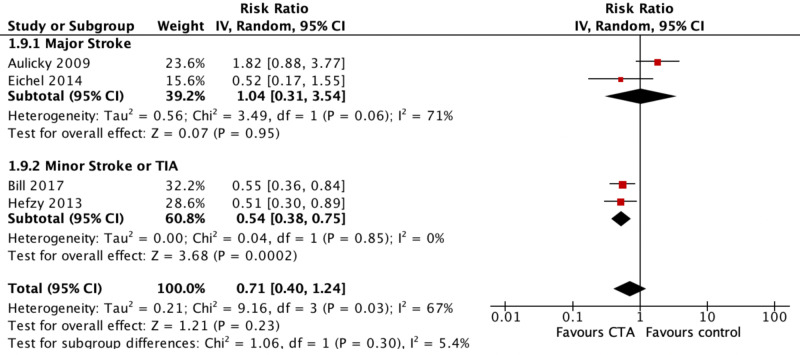
Mortality for CTA vs non-CTA, long-term outcomes Three pooled effect estimates (diamonds) are shown: one each for studies in major stroke patients, minor stroke/TIA patients, and their combination (total). Data are expressed as risk ratios with 95% confidence intervals, using generic inverse-variance random-effects models. Interstudy heterogeneity was tested using the Cochran Q statistic (chi-square) at a significance level of P < 0.10 and quantified using the *I^2^* statistic. CTA: Computed tomography angiography; TIA: Transient ischemic attack.

**Figure 16:**
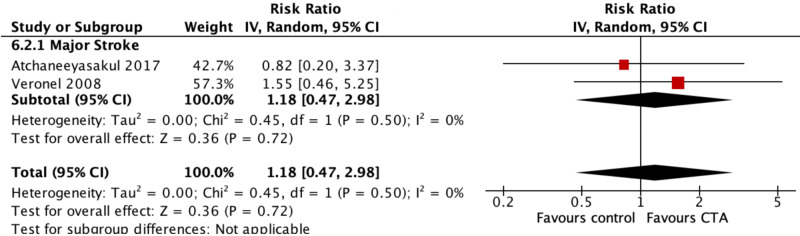
Disability for CTA vs NCCT, in-hospital outcomes The pooled effect estimates (diamonds) are shown for studies in major stroke patients and the total. There were no studies in minor stroke/TIA. Data are expressed as risk ratios with 95% confidence intervals, using generic inverse-variance random-effects models. Interstudy heterogeneity was tested using the Cochran Q statistic (chi-square) at a significance level of P < 0.10 and quantified using the *I^2^* statistic. CTA: Computed tomography angiography; NCCT: Non-contrast computed tomography; TIA: Transient ischemic attack.

**Figure 17:**
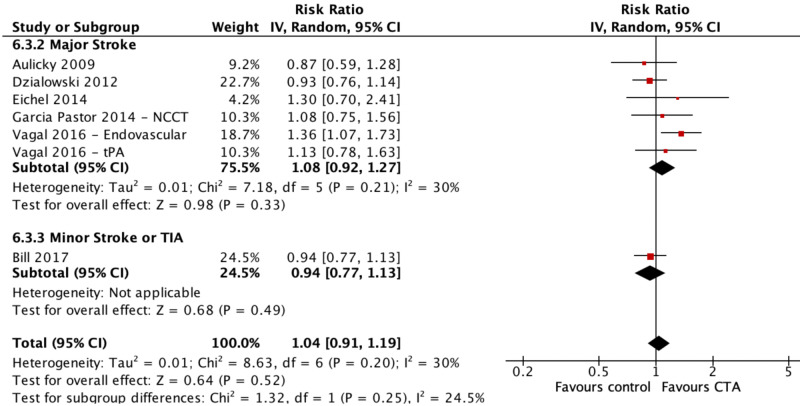
Disability for CTA vs NCCT, long-term outcomes Three pooled effect estimates (diamonds) are shown: one each for studies in major stroke patients, minor stroke/TIA patients, and their combination (total). Data are expressed as risk ratios with 95% confidence intervals, using generic inverse-variance random-effects models. Interstudy heterogeneity was tested using the Cochran Q statistic (chi-square) at a significance level of P < 0.10 and quantified using the *I^2^* statistic. CTA: Computed tomography angiography; NCCT: Non-contrast computed tomography; TIA: Transient ischemic attack.

**Figure 18:**
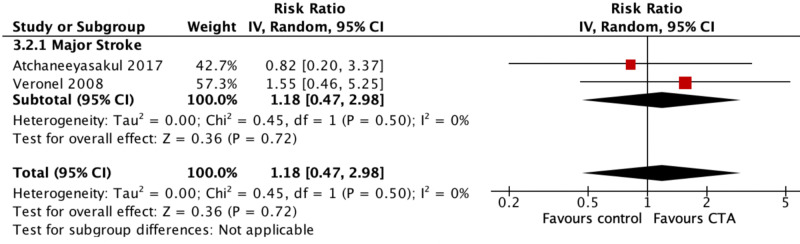
Disability for CTA vs non-CTA, in-hospital outcomes The pooled effect estimates (diamonds) are shown for studies in major stroke patients and the total. There were no studies in minor stroke/TIA. Data are expressed as risk ratios with 95% confidence intervals, using generic inverse-variance random-effects models. Interstudy heterogeneity was tested using the Cochran Q statistic (chi-square) at a significance level of P < 0.10 and quantified using the *I^2^* statistic. CTA: Computed tomography angiography; TIA: Transient ischemic attack.

**Figure 19:**
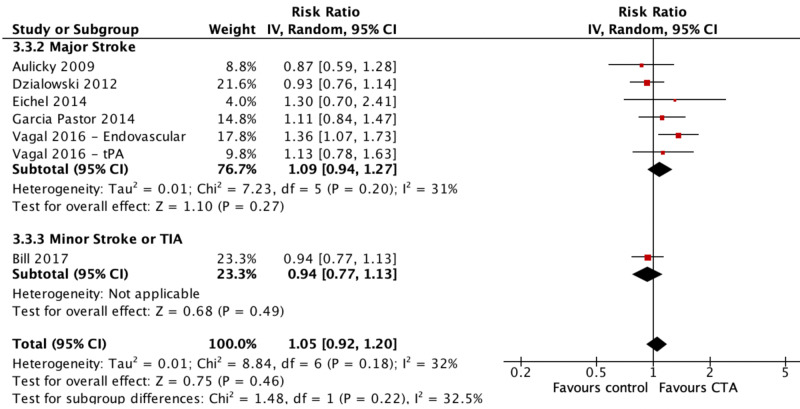
Disability for CTA vs non-CTA, long-term outcomes Three pooled effect estimates (diamonds) are shown: one each for studies in major stroke patients, minor stroke/TIA patients, and their combination (total). Data are expressed as risk ratios with 95% confidence intervals, using generic inverse-variance random-effects models. Interstudy heterogeneity was tested using the Cochran Q statistic (chi-square) at a significance level of P < 0.10 and quantified using the *I^2^* statistic. CTA: Computed tomography angiography; TIA: Transient ischemic attack.

## Appendices

**Table 4:** Search Strategy

Database: EBM Reviews - Cochrane Central Register of Controlled Trials
Search Strategy:
1 Ischemic Attack, Transient/ (636)
2 exp *Stroke/ (4210)
3 "Transient ischemic attack*".ti,ab,hw. (1924)
4 (TIA or TIAs).ti,ab,hw. (1042)
5 "Transient brainstem ischemia*".ti,ab,hw. (0)
6 "Transient cerebral ischemia*".ti,ab,hw. (18)
7 (stroke or strokes).ti,ab,hw. (40766)
8 "cerebrovascular accident*".ti,ab,hw. (9147)
9 "CVA*".ti,ab,hw. (422)
10 apoplexy.ti,ab,hw. (266)
11 ministroke*.ti,ab,hw. (0)
12 mini-stroke*.ti,ab,hw. (5)
13 "minor stroke*".ti,ab,hw. (261)
14 1 or 2 or 3 or 4 or 5 or 6 or 7 or 8 or 9 or 10 or 11 or 12 or 13 (45051)
15 exp Tomography, X-Ray Computed/ (4661)
16 exp Angiography/ (6994)
17 15 and 16 (635)
18 "computed tomography angiograph*".ti,ab,hw. (544)
19 "CT angiograph*".ti,ab,hw. (742)
20 CTA*.ti,ab,hw. (814)
21 17 or 18 or 19 or 20 (1915)
22 14 and 21 (297)
Database: Embase Classic+Embase <1947 to 2019 March 06>
Search Strategy:
1 transient ischemic attack/ (35745)
2 exp *cerebrovascular accident/ (74931)
3 transient ischemic attack*.tw. (15992)
4 (TIA or TIAs).tw. (17605)
5 "Transient brainstem ischemia*".tw. (9)
6 "Transient cerebral ischemia*".tw. (1566)
7 (stroke or strokes).tw. (353025)
8 "cerebrovascular accident*".tw. (10379)
9 "CVA*".tw. (8369)
10 apoplexy.tw. (4054)
11 "brain vascular accident*".tw. (14)
12 Ministroke*.tw. (15)
13 "Mini-stroke*".tw. (53)
14 "minor stroke*".tw. (2548)
15 1 or 2 or 3 or 4 or 5 or 6 or 7 or 8 or 9 or 10 or 11 or 12 or 13 or 14 (400147)
16 computed tomographic angiography/ (48403)
17 "computed tomography angiograph*".tw. (9270)
18 "CT angiograph*".tw. (17232)
19 CTA*.tw. (26974)
20 16 or 17 or 18 or 19 (72701)
21 15 and 20 (7907)
22 exp animal/ not human/ (5334415)
23 21 not 22 (7879)
24 limit 23 to embase (4772)
Database: All Ovid Medline <1946 - present>
Search Strategy:
1 Ischemic Attack, Transient/ (19664)
2 exp *Stroke/ (88971)
3 "Transient ischemic attack*".tw. (10410)
4 (TIA or TIAs).tw. (8167)
5 "transient brainstem ischemia*".tw. (6)
6 "Transient cerebral ischemia*".tw. (1231)
7 (stroke or strokes).tw. (220688)
8 cerebrovascular accident*.tw. (6327)
9 CVA*.tw. (3913)
10 apoplexy.tw. (2894)
11 "brain vascular accident*".tw. (10)
12 Ministroke*.tw. (12)
13 "Mini-stroke*".tw. (37)
14 "minor stroke*".tw. (1454)
15 1 or 2 or 3 or 4 or 5 or 6 or 7 or 8 or 9 or 10 or 11 or 12 or 13 or 14 (264822)
16 exp Tomography, X-Ray Computed/ (398998)
17 exp Angiography/ (233119)
18 16 and 17 (43896)
19 computed tomography angiograph*.tw. (6510)
20 CT angiograph*.tw. (9641)
21 CTA*.tw. (19837)
22 18 or 19 or 20 or 21 (65749)
23 15 and 22 (4933)
24 exp Animals/ not Humans/ (4553712)
25 23 not 24 (4910)
